# How significant is cost-shifting behavior under the diagnosis intervention packet payment reform? Evidence from the coronary heart disease market

**DOI:** 10.3389/fpubh.2024.1431991

**Published:** 2024-11-20

**Authors:** Huawei Tan, Xueyu Zhang, Shengxian Bi, Yingchun Chen, Dandan Guo

**Affiliations:** Department of Health Management, School of Medicine and Health Management, Tongji Medical College, Huazhong University of Science and Technology, Wuhan, China

**Keywords:** payment reform, regional global budget, diagnosis-intervention packet, hospital behavior, CHD, cost-containment, cost-shifting, China

## Abstract

**Background:**

Controlling the growth of inpatient costs presents a major challenge in China’s healthcare system. China introduced a new case-based payment method, the “Diagnosis Intervention Packet” (DIP), to address the surge in hospitalization expenses. However, the influence of DIP payment reform on cost shifting among coronary heart disease (CHD) inpatients remains unclear.

**Methods:**

This study focused on Zunyi, a national pilot city for DIP, utilizing inpatient claim data to assess the effects of DIP payment reform. We analyzed the influence on total health expenditures (THE), individual payments excluding reimbursement (IPER), proportion of IPER, copayments for category-B, proportion of copayments for category-B, copayments for category C, and proportion of copayments for category C per case for CHD inpatient.

**Results:**

Results indicate a significant reduction in THE per case for CHD inpatients after the DIP reform (*β* = −0.1272, *p* < 0.01). Increases in cost shifting were observed in IPER (*β* = 0.1080, *p* < 0.05), the proportion of IPER (*β* = 0.0551, *p* < 0.01), copayments for category B (*β* = 0.2392, *p* < 0.01), and the proportion of copayments for category B (*β* = 0.0295, *p* < 0.01), along with the proportion of copayments for category C (*β* = 0.0255, *p* < 0.01). However, the copayments for category C did not significantly change. Notable variations in the effects of cost control and shifting were observed across different hospital categories, teaching statuses, hospital grades, and ownership types.

**Conclusion:**

The DIP reform significantly reduced the THE per case for CHD inpatients, while shifting in-policy expenditures to IPER, particularly with a greater shift intensity in the proportion of Class B compared with the proportion of Class C.

## 1 Introduction

The escalation of health spending has garnered global attention due to concerns about the affordability and accessibility of health services (1). Inappropriate incentive mechanisms for healthcare providers are a primary driver of this spending increase (2). Health insurance payment reform is widely regarded as the most critical “lever” for curbing health expenditure growth (3). Developed and developing countries are exploring effective payment methods for healthcare providers that reduce the inherent motivation to increase service volume typical of fee-for-service (FFS) systems and realign provider incentives, thereby controlling the inflation of health expenditures (4).

In China, the rate of health spending inflation is particularly alarming. In 2010, the national health expenditures were CNY 1998 billion, which escalated to CNY 7684.4 billion by 2021, marking a 2.84-fold increase (5). Inpatient expenditures are a major driver of this inflation. The proportion of inpatient expenditures in total national health expenditures is expected to rise from 46 to 53% from 2015 to 2035 (6). FFS is considered the main catalyst for the rapid increase in inpatient expenditures in China (7).

To control the growth of inpatient expenses, China has adopted a “dual-track” system of diagnosis-related group (DRG) and diagnosis intervention packet (DIP) payments to replace the traditional FFS system (8). DIP, an innovative inpatient service payment method, operates under a regional global budget (RGB) and is distinguished from DRGs by its more uniform resource consumption within groups, simpler design, dynamic grouping, and reimbursement values that more accurately reflect real-world treatment paths and costs, making it easier to implement (9). Consequently, DIP has been rapidly advanced across mainland China, with twice as many cities implementing DIP compared with those adopting DRG (10).

In recent years, the cost-containment effects of DIP have increasingly come under scrutiny, though findings remain mixed. Lai et al. reported that after the DIP payment reform, the total health expenditures (THE) and drug expenditures per case for inpatients decreased (9). Ding et al. observed a significant decline in THE per case in secondary and tertiary hospitals following the implementation of DIP (11). Ying et al. noted an increase in THE per case after the DIP payment reform (12). These studies predominantly focus on regions in the developed eastern cities, with scant evidence from central and western China. Furthermore, previous studies often form treatment and control groups from local inpatients and non-local inpatients to assess the cost-containment effects of the DIP policy (9, 12). However, significant theoretical and empirical research suggests that following DRG reforms, hospitals may engage in opportunistic behavior, shifting inpatient expenditures to uninsured patients (13–16). Thus, research designs that utilize non-local inpatients as control groups may encounter endogeneity issues.

In China, cardiovascular diseases (CVD) are the leading cause of death among urban and rural residents. In 2020, CVD accounted for 48.00% of rural deaths and 45.86% of urban deaths (17). Coronary heart disease (CHD) ranks third in mortality rates among the various types of CVD. Consequently, CHD represents a significant source of disease burden and is a major driver of the growth in hospitalization expenditures among Chinese residents (18).

In this work, we analyze inpatient claim data from Zunyi, a national pilot city for the DIP in western China, using a quasi-natural experimental design. We compare Zunyi with Guiyang and Chongqing—two cities where Zunyi’s insured residents frequently seek medical treatment but which have not yet implemented DRG or DIP reforms. These cities serve as control groups, allowing us to evaluate the cost-containment and cost-shifting effects of DIP payment reform on CHD. This method effectively circumvents the endogeneity problem typically associated with cost shifting. Our study thus contributes to and broadens the existing literature on the cost-shifting behaviors of prospective payment systems (PPS) in middle- and low-income countries.

## 2 Institutional background

### 2.1 Overview of the DIP payment reform in Zunyi

Zunyi City, located in the southwest region of China adjacent to Guiyang City and Chongqing, reported a GDP of CNY 440.1 billion in 2022, with a registered population of 8.2636 million and a resident population of 6.5965 million by the end of the year. Zunyi administers 14 districts and counties. In October 2021, Zunyi implemented the DIP payment reform across 70 secondary hospitals and 10 tertiary hospitals within the city.

The DIP payment system in Zunyi features a “settlement separation” mechanism, where settlements are divided into patient-side and insurance-side components. With regard to the inpatients in Zunyi’s secondary and tertiary hospitals, settlements are made on an FFS basis on the patient side, while the insurance side is settled according to DIP point volume. Regarding residents insured in Zunyi who seek medical treatment outside the city, both patients and insurers employ the FFS system.

On the patient side, the FFS items are categorized into three classes: Classes A, B, and C. Class A is fully covered by insurance; Class B requires an upfront payment of 10% with the remaining 90% covered by insurance; Class C is not covered by insurance at all. In China’s basic medical insurance system, individual payments excluding reimbursement (IPER) include copayments for category B (i.e., upfront payment of 10% of Class B) and category C, along with deductibles.

Guiyang and Chongqing are the two major cities where Zunyi’s insured residents frequently seek medical treatment and have not yet initiated DRG or DIP reforms. Accordingly, the insured members from Zunyi using different payment methods inside and outside the city provide an excellent quasi-natural experiment for this study.

### 2.2 Hospital response after DIP payment reform

Theoretically, the DIP payment system is guided by yardstick competition theory and PPS, which aim to modify hospital behavior by adjusting financial incentives (19, 20). Specifically, the DIP payment alters hospital financial incentives in terms of risk, intensity, stability, and expectations as well as surplus management strategies, thereby influencing hospital behavior.

First, the RGB enforces a stringent budget constraint at the regional level, preventing deficits in health insurance funds and transferring the risk of these deficits to hospitals (21). RGB transitions from the previous single-hospital global budget approach to a city-wide global budget, altering the predictability and risk associated with financial incentives for hospitals.

Second, under DIP payment, the actual payment for treated cases is flexible and not pre-fixed, ensuring that total hospital compensation stays within a pre-determined regional health budget. The design of the DIP point value fluctuation mechanism makes each hospital’s annual reimbursement depend not only on its own service volume but also on the service volume of other hospitals in the region. This fluctuation in point value alters the predictability and stability of financial incentives for hospitals.

Third, hospitals are incentivized to increase their total points to prevent a devaluation of these points at year-end settlements due to the design of point value fluctuations. This point competition alters the intensity and stability of incentives for medical institutions.

Fourth, DIP, a prepaid system, encourages hospitals to transition from revenue centers to cost centers (22). Although the revenue for each patient in a DIP group is variable, the incentive to keep total expenses below the anticipated payment persists. Therefore, hospitals are strongly motivated to provide more cost-effective services to maximize the surplus obtained from the insurance provided for DIP cases.

Under the financial incentives of DIP, hospitals primarily strive to maximize revenue or minimize costs through three behaviors: point competition, cost control, and cost shifting, particularly in China’s competitive healthcare environment (23, 24). Cost shifting is primarily achieved through patient and cost transfers, including moving inpatients to outpatient settings, upcoding, and shifting inpatient costs to outpatient services, uninsured individuals, and from within the insurance policy to IPER. Under the DIP settlement separation mechanism, cost-shifting behaviors are likely to occur on the patient side, aimed primarily at maximizing revenue per hospitalized patient. Cost-shifting behaviors typically involve shifting coverage of reimbursement to IPER to lower the actual reimbursement ratio for patients, thereby increasing the end-of-year settlement point value. This creates a substitution effect between coverage of reimbursement and IPER. The absence of real-world cost data for specific diseases further encourages these behaviors. Nonetheless, the internal shifts in IPER, specifically within category-B and category-C copayments, are not well-documented and remain an area for further investigation.

## 3 Methods

### 3.1 Data sources

Our study utilizes three primary data sources:

1. Inpatient claim data: This dataset, obtained from the Zunyi Health Insurance Bureau, includes patient demographics (age, gender, and insured classifications), visit dates (admission and discharge dates), disease characteristics [main diagnosis, complications, surgery status, and length of hospital stay (LOS)], hospital characteristics (hospital name, code, and grade), and hospitalization expenditures (THE, individual payments excluding reimbursement, copayments for category B, copayments for category C, and deductibles).
2. Supplementary hospital characteristics in Zunyi: Data on additional characteristics of hospitals in Zunyi, such as category, ownership, and teaching status, were acquired from the Zunyi Health Commission.
3. Supplementary hospital characteristics in Guiyang and Chongqing: Information regarding hospital category, ownership, and teaching status was collected from the official hospital websites or local government websites of Guiyang and Chongqing, primarily through manual searches.

#### 3.1.1 Selection criteria for inpatients

Inpatients for CHD were identified using the main diagnosis codes from the ICD-10 (I25.104, Z03.501, I25.102, and I25.103). All identifiable patient information, such as names, ID numbers, addresses, and inpatient numbers, was removed before the data were made available to the research team to ensure privacy.

### 3.2 Measurements

#### 3.2.1 Outcome variables

This study primarily assesses the cost-shifting effects of the DIP. The IPER and copayments served as proxies for cost-shifting. Additionally, the copayments for category-B, the proportion of copayments for category-B, the copayments for category-C, and the proportion of copayments for category-C were computed to analyze specific pathways of cost-shifting.

#### 3.2.2 Explanatory variable

In this study, all CHD inpatients in secondary and tertiary hospitals in Zunyi were considered the treatment group, while those in Guiyang’s and Chongqing’s secondary and tertiary hospitals served as the control group. October 2020 to September 2021 and October 2021 to December 2022 were set as the pre- and post-policy intervention periods, respectively, to exclude the impact of the COVID-19 pandemic.

#### 3.2.3 Control variables

Control variables were constructed across three dimensions: patient demographics, hospital characteristics, and disease characteristics. Patient demographics included age, gender (female = 0, male = 1), and insured classifications, with urban and rural residents’ basic medical insurance (URRBMI) coded as 0 and Urban Employee Medical Insurance (UEMI) as 1. Hospital characteristics encompassed hospital category (general hospital [GH] = 0, traditional Chinese medicine [TCM] hospital = 1), ownership (public = 0, private = 1), teaching status (teaching = 0, non-teaching = 1), and Hospital grade (Secondary hospital = 0, Tertiary hospital = 1). Disease characteristics include the presence of complications (no = 0, yes = 1) and LOS.

### 3.3 Statistical analysis

#### 3.3.1 Propensity score matching method (PSM)

China has yet to establish a mandatory gatekeeper system, allowing patients the freedom to choose hospitals. Additionally, the DIP payment reform is not a randomized clinical intervention but a non-randomized experiment, which does not conform to the assumptions of randomness and homogeneity (25). Consequently, presuming that inpatients are randomly assigned to control and treatment groups is impractical. We utilized the PSM method to mitigate potential sample selection biases between the control and the treatment groups. This method involved second-nearest neighbor matching, allowing for individual matching by disease. The model is shown in Equation 1.


(1) $$\mathrm{Logit}\left(D_{i}=1\right)=\alpha +\beta X_{i}+\varepsilon_{i}$$

where *D_i_* is a dummy variable for the implementation of the DIP payment reform: coded as 1 for CHD inpatients within Zunyi and 0 for those hospitalized outside Zunyi (in Guiyang and Chongqing). *X_i_* includes matching variables, such as inpatient age, gender, hospital category, hospital ownership, hospital teaching status, and hospital grade, whether there are comorbidities or complications, and length of hospital stay. Based on the estimated propensity scores, we use the third-nearest neighbor matching method to match treatment and control group samples annually.

#### 3.3.2 Difference-in-differences (DID) method

Our main empirical strategy is based on a DID analysis with fixed effects for inpatient, hospital, and month (25). We construct the following regression model based on the method used by Lai et al. (9). The model is shown in Equation 2.


(2) Yiht=α+βEventiht+θXiht+μt+νh+εiht

where *Event_iht_* is the key explanatory variable. This dummy variable represents the status of the DIP payment reform. Moreover, this variable equals one if a CHD inpatient *i* comes from a hospital within Zunyi City and was discharged after January 1, 2022; otherwise, it is zero. The coefficient *β* captures the average treatment effect of the DIP payment reform. The dependent variable *Y_iht_* represents the THE, IPER, and proportion of IPER for a specific CHD inpatient *i* in hospital *h* and at time *t*. All these expenditure variables are estimated in logs due to the skewed distribution of health expenditure. Control variables *X_iht_* include patient age, gender, hospital category, hospital ownership, hospital teaching status, hospital grade, whether there are comorbidities or complications, and length of hospital stay. *μ_t_* represents a month dummy variable to control for flexible time effects. *ν_h_* represents a series of hospital dummy variables to control for unobserved time-variant heterogeneity between hospitals. *ε_iht_* represents the robust error terms at the hospital-month level. All analyses are performed using Stata 17.0 for Windows, with significance levels set at 0.1, 1, and 5%.

### 3.4 Parallel trend test

A prerequisite for the double-difference approach is that the trends in total hospitalization expenses, IPER, and the proportion of IPER in the treatment and control groups were parallel before the DIP reform. We use an event study methodology to test for parallel trends in the treatment and control groups following the approach of D’Haultfoeuille et al. (26). The model is shown in Equation 3.


(3) $$Log\left(Yit\right)=\alpha +\overset{14}{\underset{n=-14}{\sum }}\mathrm{\beta nDI}D_{\mathrm{it}}^{n}+\mathrm{\gamma Xit}+\mu i+\lambda i+\varepsilon it$$

DID is generated as a relative year policy variable with reference to the year of the DIP reform, where Zunyi variable equals one, while the variables for Chongqing and Guiyang are always zero. The month of the DIP reform is set as the baseline time for the event study; *β_n_* is the regression coefficient relative to the baseline year; *X_it_* includes control variables, such as patient age, gender, hospital type, hospital economic ownership, hospital level, whether it is a teaching hospital, presence of comorbidities, and length of hospital stay. If the pre-reform coefficients *β_n_* are not significantly different from zero, then the assumption of parallel trends is met, and the estimated results of *β_n_* within the 95% confidence interval can be plotted.

### 3.5 Sensitivity analysis

This study utilizes three methods to check for robustness:

1. Alternative matching methods: We replaced the fifth-nearest neighbor matching method with radius and kernel matching, followed by re-estimating the DID after matching the samples.
2. Influence of outliers: In the baseline regression, the dependent variables and the length of hospital stays were Winsorized at the 1 and 99% percentiles, replacing values above the 99% percentile and below the 1% percentile, respectively. Subsequently, we re-estimated DID.
3. Placebo test: We conducted a random experiment at the reform time-hospital level by “randomly” selecting DIP and generating reform times. This process was repeated 500 times to enhance the power of the placebo test. We plotted a distribution chart of the estimated DID coefficients.

### 3.6 Heterogeneity analysis

Previous theoretical and empirical literature extensively documents that different types of hospitals exhibit diverse response strategies to PPS (11, 16, 27–30). Accordingly, this study explores the heterogeneity of the cost-containment and cost-shifting effects of DIP across various hospital dimensions, including category, ownership, teaching status, and grade.

## 4 Results

### 4.1 Descriptive statistics

Table 1 presents the results of the descriptive statistical analysis. The final sample comprised 54,685 inpatients from 180 hospitals. We observed significant differences between the treatment and the control groups in several metrics, including THE, IPER, and copayments for categories B and C, as well as the proportion of IPER and the proportion of category B copayments among CHD inpatients. Although the patient characteristics were similar between groups, notable differences were evident in hospital characteristics.

**Table 1:** Summary statistics.

Variables	Observations	Mean			SD	Min	Median	Max
		Full sample	Treatment group	Control group				
THE	54,685	8558.536	8325.175	17018.182	11160.527	917.940	4827.650	64682.84
IPER	54,685	1431.611	1368.609	3715.520	2937.001	53.130	481.630	18312.14
Copayments for category-B	54,685	544.283	526.927	1173.458	1034.018	25.120	211.430	6263.29
Copayments for category-C	54,685	868.478	827.392	2357.889	1993.025	0.000	249.240	12866.48
Proportion of IPER	54,685	0.121	0.119	0.198	0.081	−2.528	0.104	0.874
Proportion of copayments for category-B	54,685	0.051	0.050	0.064	0.024	−0.004	0.047	0.595
Proportion of copayments for category-C	54,685	0.070	0.069	0.134	0.076	−2.528	0.054	0.864
Gender	54,685	0.505	0.504	0.534	0.500	0.000	1.000	1.000
Age group	54,685	3.919	3.924	3.723	0.664	1.000	4.000	5.000
Hospital grade	54,685	0.402	0.390	0.847	0.490	0.000	0.000	1.000
Hospital category	54,685	0.216	0.218	0.138	0.458	0.000	0.000	2.000

### 4.2 Propensity score matching results

#### 4.2.1 Balance test

After matching, we performed a balance test to evaluate the effectiveness of the matching process. As shown in Table 2, the *p*-values for all covariates after matching exceeded 5%. Furthermore, the absolute values of the standard deviations for the matching variables were all below 10%. This evidence supports the effectiveness of PSM.

**Table 2:** Balance test of PSM for treatment and control groups.

Variable		Mean		%bias	t-test	
		Treated	Control		t	p
Gender	Unmatched	0.5282	0.5476	−3.90	−0.93	0.351
	Matched	0.5248	0.5316	−1.40	−1.34	0.179
Age group	Unmatched	3.9251	3.6403	44.7	7.67	0.000
	Matched	3.9235	3.9443	−3.30	0.76	0.445
Hospital grade	Unmatched	1.4084	1.8554	−104.60	−21.88	0.000
	Matched	1.4137	1.4067	1.60	1.38	0.167
Hospital category	Unmatched	0.7860	0.6701	26.30	6.72	0.000
	Matched	0.7866	0.7840	0.60	0.61	0.540
Ln (LOS)	Unmatched	2.0204	2.0339	−2.70	−2.58	0.010
	Matched	2.0103	2.0141	−0.70	−0.16	0.872

#### 4.2.2 Kernel density plot

**Figure 1:**
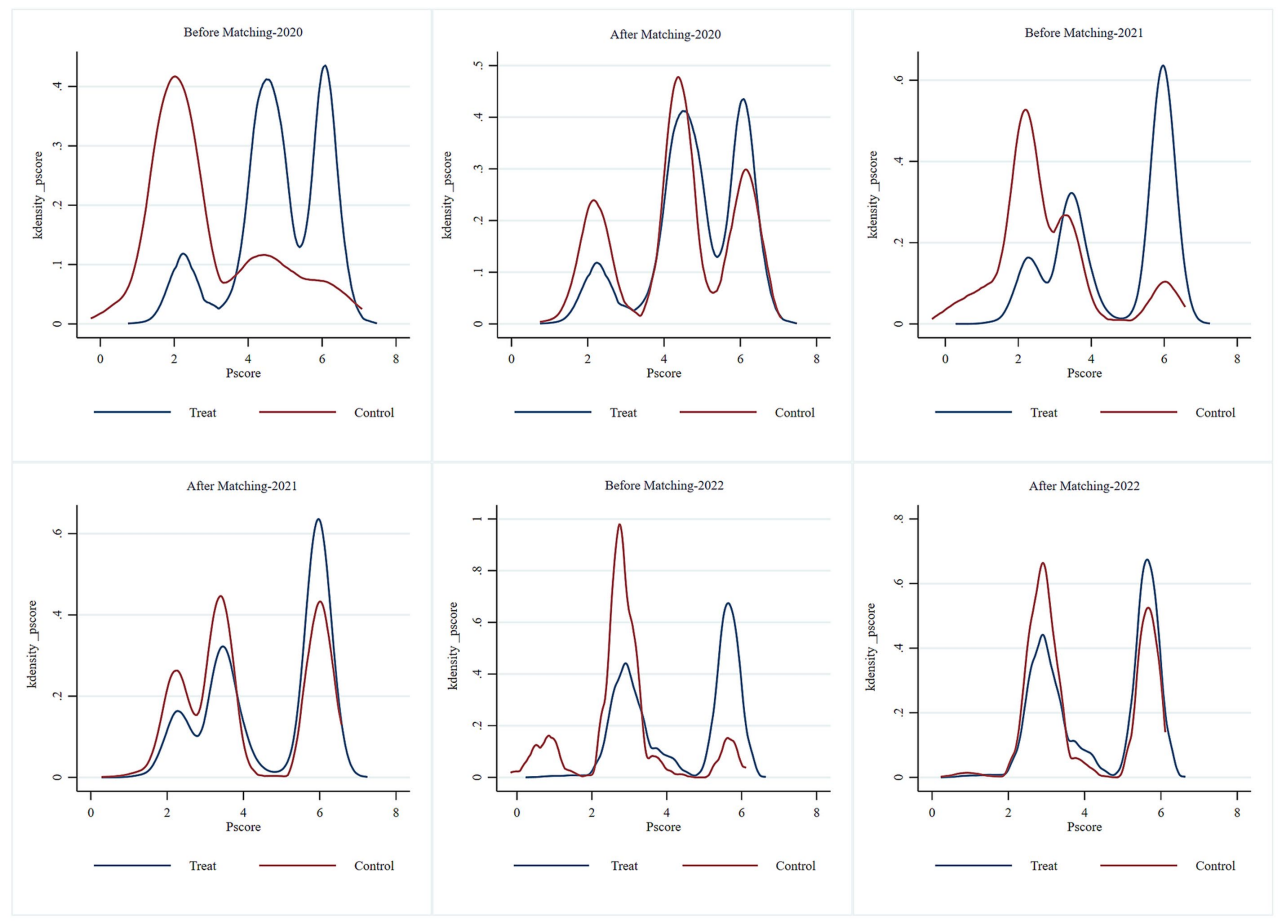
Kernel density plot.

Figure 1 illustrates the distribution of the kernel density functions for THE before and after PSM. Prior to matching, a marked difference was observed in the probability distribution of the propensity scores between the control and experimental groups, with limited overlap in their common support domain. After matching, the probability distributions of the propensity scores for both groups nearly overlapped, indicating high-quality matching. This finding demonstrates that the study satisfies the common support condition assumption.

### 4.3 Parallel trend test

**Figure 2:**
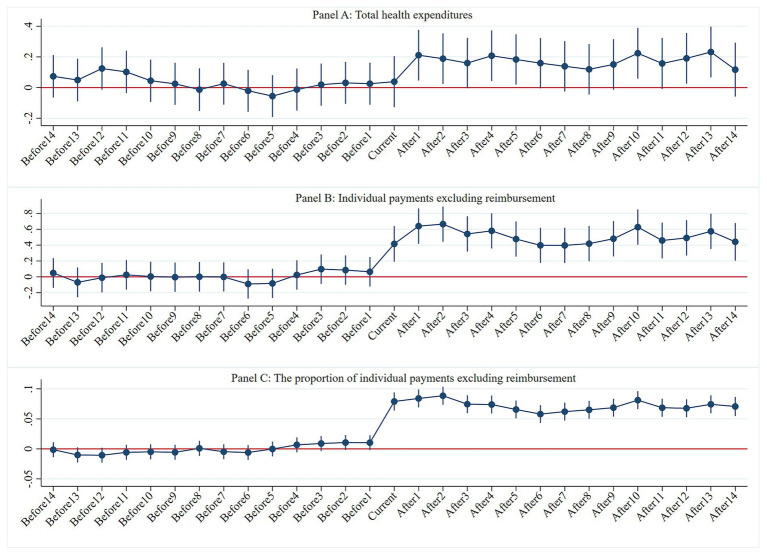
Parallel trends test. Panel A: Total health expenditures, Panel B: Individual payments excluding reimbursement, Panel C: The proportion of individual payments excluding reimbursement.

Figure 2 displays the event study results for the 14 periods before and after the treatment. Prior to the DIP reform, no significant differences were observed in THE, IPER, and the proportion of IPER between the pilot and non-pilot areas. Following the DIP reform, the effects of cost control and cost shifting associated with DIP began to gradually emerge. The findings confirm that, before the pilot’s implementation, the treatment and control groups met the criteria for the parallel trends test, validating the methodological approach.

### 4.4 Baseline regression

Table 3 presents the baseline regression results of the model. Columns 1, 2, and 3 illustrate the effects of the DIP payment reform on THE, IPER, and the proportion of IPER per case for CHD inpatients, respectively. Specifically, after the DIP payment reform, the THE per case for CHD inpatients decreased by 11.94% (=1-exp(−0.1272), *p* < 0.01), while IPER increased by 11.40% (=exp(0.1080) − 1, *p* < 0.05), and the proportion of IPER increased by 5.51% (*p* < 0.01). These results indicate that the DIP reform reduced significantly the THE per case for CHD inpatients, yet simultaneously resulted in a significant increase in IPER.

**Table 3:** Baseline regression.

Variables	ln (THE)	ln (IPER)	Proportion of IPER
	(1)	(2)	(3)
DIP payment	−0.1272***	0.1080**	0.0551***
	(0.0389)	(0.0540)	(0.0042)
Gender	0.1028***	0.1337***	0.0053***
	(0.0062)	(0.0086)	(0.0007)
Age group	−0.1191***	−0.1748***	−0.0054***
	(0.0048)	(0.0067)	(0.0005)
Hospital grade	0.1433***	0.0947***	−0.0065***
	(0.0076)	(0.0106)	(0.0008)
Hospital category	−0.2063***	−0.4672***	−0.0268***
	(0.0072)	(0.0100)	(0.0008)
Hospital ownership	−0.0426***	0.0243	0.0091***
	(0.0126)	(0.0175)	(0.0013)
Hospital teaching status	−0.8252***	−1.2131***	−0.0548***
	(0.0099)	(0.0138)	(0.0011)
Comorbidities or complications	−0.0870***	−0.1958***	−0.0117***
	(0.0189)	(0.0263)	(0.0020)
Length of hospital stay	0.7845***	0.8501***	0.0107***
	(0.0062)	(0.0085)	(0.0007)
Constant	8.3894***	7.1136***	0.2688***
	(0.0369)	(0.0513)	(0.0040)
Month FE	Yes	Yes	Yes
Hospital FE	Yes	Yes	Yes
Observations	53,388	53,300	53,388
R-squared	0.3640	0.3358	0.1211

Robust standard errors in parentheses, ***p < 0.001, **p < 0.01, *p < 0.05.

### 4.5 Sensitivity analysis

We conducted robustness tests by changing the matching method and removing outliers to enhance the robustness of our conclusions. Table 4 presents the regression results after applying radius matching, kernel matching, and outlier removal techniques. The results demonstrate that the regression outcomes remain consistent with the baseline regression results even after varying the matching methods and addressing outliers. This consistency reinforces the robustness of our study findings.

**Table 4:** Sensitivity analysis.

Variables	ln (THE)			ln (IPER)			Proportion of IPER		
	Radius matching	Kernel matching	Outlier removal	Radius matching	Kernel matching	Outlier removal	Radius matching	Kernel matching	Outlier removal
	(1)	(2)	(3)	(4)	(5)	(6)	(7)	(8)	(9)
DIP payment	−0.1306***	−0.1304***	−0.1362***	0.0937*	0.1033*	0.0984*	0.0528***	0.0547***	0.0497***
	(0.0393)	(0.0389)	(0.0383)	(0.0546)	(0.0539)	(0.0535)	(0.0042)	(0.0042)	(0.0035)
Constant	8.3814***	8.3842***	8.3468***	7.0958***	7.1063***	7.0494***	0.2669***	0.2683***	0.2641***
	(0.0372)	(0.0369)	(0.0364)	(0.0517)	(0.0512)	(0.0510)	(0.0040)	(0.0040)	(0.0033)
Control variables	Yes	Yes	Yes	Yes	Yes	Yes	Yes	Yes	Yes
Month FE	Yes	Yes	Yes	Yes	Yes	Yes	Yes	Yes	Yes
Hospital FE	Yes	Yes	Yes	Yes	Yes	Yes	Yes	Yes	Yes
Observations	53,330	53,337	53,406	53,242	53,249	53,406	53,330	53,337	53,406
R-squared	0.3632	0.3631	0.3606	0.3347	0.3352	0.3280	0.1198	0.1214	0.1702

Robust standard errors in parentheses, ***p < 0.001, **p < 0.01, *p < 0.05.

### 4.6 Placebo test

**Figure 3:**
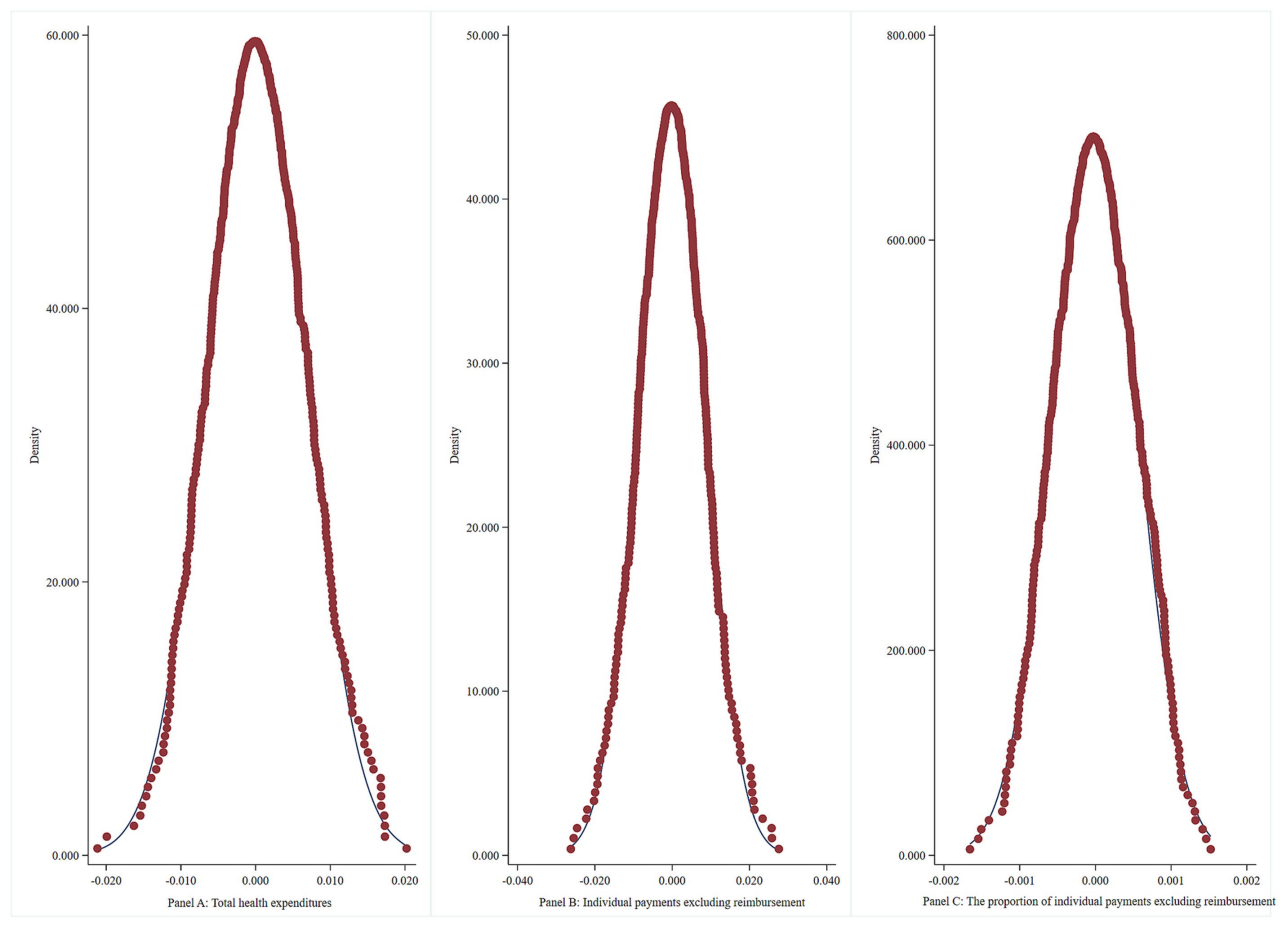
Placebo test.

The results of the placebo test (Figure 3) show that the distribution of the coefficient density estimates from the random process clusters around zero. This notion indicates no significant omitted variable issues within the model setup, thereby confirming the robustness of our conclusions.

### 4.7 Heterogeneity analysis

#### 4.7.1 Hospital category

Table 5 displays the results of the heterogeneity test examining the cost-containment and cost-shifting effects of the DIP on different categories of hospitals. Columns 1, 3, and 5 detail the effects of the DIP payment reform on THE, IPER, and the proportion of IPER per case for CHD inpatients in general hospitals. After the DIP payment reform, the THE per case for CHD inpatients in general hospitals decreased by 10.60% (=1 − exp(−0.1121), *p* < 0.05), IPER increased by 14.22% (=exp(0.1330) − 1, *p* < 0.05), and the proportion of IPER increased by 5.75% (*p* < 0.01).

**Table 5:** The cost-containment effects of DIP across different categories of hospitals.

Variables	ln (THE)		ln (IPER)		Proportion of IPER	
	General hospital	Traditional Chinese medicine hospital	General hospital	Traditional Chinese medicine hospital	General hospital	Traditional Chinese medicine hospital
	(1)	(2)	(3)	(4)	(5)	(6)
DIP payment	−0.1121**	−0.2620	0.1330**	−0.4276*	0.0575***	−0.0211
	(0.0492)	(0.1741)	(0.0653)	(0.2255)	(0.0047)	(0.0140)
Constant	9.4566***	8.0434***	8.3231***	5.4556***	0.2944***	0.0651***
	(0.0466)	(0.1500)	(0.0618)	(0.1943)	(0.0044)	(0.0121)
Control variables	Yes	Yes	Yes	Yes	Yes	Yes
Month FE	Yes	Yes	Yes	Yes	Yes	Yes
Hospital FE	Yes	Yes	Yes	Yes	Yes	Yes
Observations	42,903	10,485	42,816	9,540	42,903	9,541
R-squared	0.1616	0.0938	0.1795	0.0855	0.0885	0.0319

Robust standard errors in parentheses, ***p < 0.001, **p < 0.01, *p < 0.05.

Columns 2, 4, and 6 show the effects of the DIP payment reform on THE, IPER, and the proportion of IPER per case for CHD inpatients in traditional Chinese medicine hospitals. Specifically, the THE per case decreased by 23.05% (=1 − exp(−0.2620), *p* > 0.05), IPER decreased by 34.79% (*p* < 0.05), and the proportion of IPER decreased by 2.11% (*p* > 0.05).

#### 4.7.2 Hospital grade

Table 6 details the heterogeneity test results for the cost-containment and cost-shifting effects of DIP on different grades of hospitals. Columns 1, 3, and 5 present the effects on THE, IPER, and the proportion of IPER per case for CHD inpatients in secondary hospitals. Specifically, the THE per case in secondary hospitals showed a rising trend, but changes were not statistically significant (*p* > 0.05). However, the IPER increased by 63.36% (=exp(0.4908) − 1, *p* < 0.05), and the proportion of IPER increased by 6.18% (*p* < 0.01).

**Table 6:** The cost-containment effects of DIP across different grades of hospitals.

Variables	ln (THE)		ln (IPER)		Proportion of IPER	
	Secondary hospital	Tertiary hospital	Secondary hospital	Tertiary hospital	Secondary hospital	Tertiary hospital
	(1)	(2)	(3)	(4)	(5)	(6)
DIP payment	0.1488	−0.2291***	0.4908***	−0.0599	0.0618***	0.0497***
	(0.1045)	(0.0543)	(0.1378)	(0.0718)	(0.0091)	(0.0055)
Constant	8.6514***	9.7001***	6.8963***	8.3599***	0.1976***	0.2630***
	(0.0965)	(0.0511)	(0.1273)	(0.0676)	(0.0084)	(0.0052)
Control variables	Yes	Yes	Yes	Yes	Yes	Yes
Month FE	Yes	Yes	Yes	Yes	Yes	Yes
Hospital FE	Yes	Yes	Yes	Yes	Yes	Yes
Observations	31,481	21,907	31,443	21,857	31,481	21,907
R-squared	0.0178	0.1683	0.0600	0.2242	0.0661	0.1269

Robust standard errors in parentheses, ***p < 0.001, **p < 0.01, *p < 0.05.

Columns 2, 4, and 6 present the effects on tertiary hospitals. Specifically, the THE per case in tertiary hospitals decreased by 20.48% (=1 − exp(−0.2291), *p* < 0.01), while the proportion of IPER increased by 4.97% (*p* < 0.01).

#### 4.7.3 Hospital ownership

Table 7 reports the results of the heterogeneity test for the cost-containment and cost-shifting effects of DIP on hospitals of different ownerships. Columns 1, 3, and 5 show the effects on THE, IPER, and the proportion of IPER per case for CHD inpatients in public hospitals. Specifically, the THE per case in public hospitals decreased by 9.05% (=1 − exp(−0.0949), *p* < 0.05). However, the IPER increased by 18.47% (=exp(0.1695) − 1, *p* < 0.05), and the proportion of IPER increased by 5.84% (*p* < 0.01).

**Table 7:** The cost-containment effects of DIP across different ownership of hospitals.

Variables	ln (THE)		ln (IPER)		Proportion of IPER	
	Public hospital	Private hospital	Public hospital	Private hospital	Public hospital	Private hospital
	(1)	(2)	(3)	(4)	(5)	(6)
DIP payment	−0.0949**	0.1745	0.1695***	0.2769	0.0584***	0.0190
	(0.0465)	(0.1678)	(0.0617)	(0.2124)	(0.0045)	(0.0121)
Constant	9.2948***	9.1055***	8.0956***	7.1143***	0.2829***	0.1383***
	(0.0421)	(0.5075)	(0.0559)	(0.6423)	(0.0040)	(0.0366)
Control variables	Yes	Yes	Yes	Yes	Yes	Yes
Month FE	Yes	Yes	Yes	Yes	Yes	Yes
Hospital FE	Yes	Yes	Yes	Yes	Yes	Yes
Observations	49,794	3,594	49,708	3,592	49,794	3,594
R-squared	0.1743	0.0814	0.2173	0.1472	0.1179	0.1262

Robust standard errors in parentheses, ***p < 0.001, **p < 0.01, *p < 0.05.

Columns 2, 4, and 6 show the effects on private hospitals. Here, the THE, IPER, and the proportion of IPER showed an upward trend after the DIP payment reform. However, the changes were not statistically significant (*p* > 0.05).

#### 4.7.4 Teaching status of hospitals

Table 8 details the results of the heterogeneity test for the cost-containment and cost-shifting effects of DIP on hospitals based on teaching status. Columns 1, 3, and 5 show the effects on THE, IPER, and the proportion of IPER per case for CHD inpatients in teaching hospitals. Specifically, after the DIP payment reform, the THE per case in teaching hospitals increased by 16.31% (=exp(0.1511) − 1, *p* < 0.1), IPER increased by 40.33% (=exp(0.3388) −1, *p* < 0.01), and the proportion of IPER increased by 4.42% (*p* < 0.01).

**Table 8:** The cost-containment effects of DIP across different status of teaching hospitals.

Variables	ln (THE)		ln (IPER)		Proportion of IPER	
	Teaching hospitals	Non-teaching hospitals	Teaching hospitals	Non-teaching hospitals	Teaching hospitals	Non-teaching hospitals
	(1)	(2)	(3)	(4)	(5)	(6)
DIP payment	0.1511*	−0.1086*	0.3388***	0.0546	0.0442***	0.0297***
	(0.0813)	(0.0592)	(0.1098)	(0.0779)	(0.0103)	(0.0048)
Constant	7.4327***	8.8762***	4.9185***	7.2508***	0.1272***	0.1944***
	(0.1708)	(0.0531)	(0.2306)	(0.0698)	(0.0217)	(0.0043)
Control variables	Yes	Yes	Yes	Yes	Yes	Yes
Month FE	Yes	Yes	Yes	Yes	Yes	Yes
Hospital FE	Yes	Yes	Yes	Yes	Yes	Yes
Observations	8,899	44,489	8,857	44,443	8,899	44,489
R-squared	0.0406	0.0418	0.0603	0.0596	0.0668	0.0481

Robust standard errors in parentheses, ***p < 0.001, **p < 0.01, *p < 0.05.

Columns 2, 4, and 6 show the effects on non-teaching hospitals. Specifically, the THE per case in non-teaching hospitals decreased by 10.29% (=1 − exp(−0.1086), *p* < 0.1), and the proportion of IPER increased by 2.97% (*p* < 0.01).

## 5 Further analysis: pathways of cost shifting

Table 9 illustrates the effects of the DIP payment reform on various cost components for CHD inpatients, across columns (1) through (4). These columns detail the effect on copayments for category B, the proportion of copayments for category B, copayments for category C, and the proportion of copayments for category C per case. Specifically, after the implementation of the DIP payment reform, copayments for category B per case for CHD inpatients increased by 27.02% (=exp(0.2392) − 1, *p* < 0.01). Although copayments for category C per case also rose, the increase was modest at 1.85% (=exp(0.0183) − 1, *p* > 0.05). Meanwhile, the proportions of copayments for categories B and C increased by 2.95 and 2.55%, respectively (*p* < 0.01).

**Table 9:** The cost-shifting effects of DIP.

Variables	ln (copayments for category-B)	ln (copayments for category-C)	Proportion of copayments for category-B	Proportion of copayments for category-C
	(1)	(2)	(3)	(4)
DIP payment	0.2392***	0.0183	0.0295***	0.0255***
	(0.0522)	(0.0802)	(0.0012)	(0.0040)
Control variables	Yes	Yes	Yes	Yes
Constant	5.7382***	6.7846***	0.0845***	0.1843***
	(0.0496)	(0.0761)	(0.0012)	(0.0038)
Month FE	Yes	Yes	Yes	Yes
Hospital FE	Yes	Yes	Yes	Yes
Observations	53,387	53,303	53,388	53,388
R-squared	0.2897	0.2528	0.1334	0.0864

Robust standard errors in parentheses, ***p < 0.001, **p < 0.01, *p < 0.05.

## 6 Discussion

Financial incentives significantly influence the decision-making processes of healthcare providers (31). The primary goal of PPS is to mitigate the growth rate of medical insurance inpatient payments and curb overall hospital cost inflation (32). In October 2021, Zunyi City transitioned the payment method for inpatient services in secondary and tertiary hospitals from the retrospective FFS system to the prospective DIP system, a method analogous to the DRG systems adopted in France and Thailand (33, 34).

Our findings indicate that the THE per case for CHD inpatients decreased by 11.94% following the DIP payment reform. This result aligns with the findings of Lai et al. (9, 11), although our estimated marginal effects are notably higher than those reported in their broader studies encompassing various diseases. This discrepancy likely stems from our study’s specific focus on CHD, a condition known to incur higher costs than the aggregate of all diseases (18). Consequently, the cost-containment influence of DIP appears more pronounced for CHD, suggesting that DIP is particularly effective in managing expenditures for complex, higher-cost diseases compared with simpler, lower-cost conditions.

The cost-containment effects of the DIP align with our study expectations. The fixed-price system under DIP transfers treatment risks from insurers to hospitals (35). Under yardstick competition, hospitals are incentivized to minimize costs while rationally behaving, regardless of income restrictions. This situation allows hospitals the potential to profit by moderating the growth of medical insurance costs (36). Although the relative prices of different DIP disease groups are fixed, the actual prices are determined post-facto based on unit price values. This calculation depends on each hospital’s service volume, the service volumes of other hospitals, and the regional medical insurance budget. Such macro-level adjustments create an opaque environment that complicates hospitals’ ability to forecast next year’s budgets, as prices annually fluctuate with overall service volume. Without clear price signals and benchmark cost data, hospitals primarily focus on balancing their financial accounts (34). When hospitals compete to increase their total point value, the unit price per point tends to decrease.

Unlike France’s full reimbursement policies, Chinese medical insurance only covers expenditures within policy limits (37). In contrast to the DIP’s settlement separation mechanism, cost-shifting behaviors occur on the patient side, primarily aimed at maximizing revenue per hospitalization. Our study observed that the absolute and relative values of IPER significantly increased after the DIP reform. This notion indicates that hospitals have shifted costs to the patient level, creating a “balloon effect” in single-patient hospitalization expenses. This effect arises from budget gaming, pricing rules, and compensation tactics. Initially, the RGB under DIP alters the “budget gaming” rules among hospitals. Previously, the Zunyi Medical Insurance Bureau employed an individual hospital budget model similar to France’s. Under this model, hospitals shifted costs from insurance-covered services to patient-paid services to avoid annual budget overruns. Furthermore, medical institutions often adopted strategic behaviors to exceed their budgets ensure the next year’s budget size. The adoption of RGB under DIP disrupted the predictability of hospital budgets, increasing the motivation to shift costs from insurance-paid to patient-paid services. Additionally, pricing rules based on historical costs lead to “cross-subsidization,” where the case mix effect’s extent is determined by established relative prices (22). Ideally, prices should reflect the actual costs and align with broader medical system goals and outcomes; failing to do so can result in unintended consequences (38). In China, DIP points are determined based on historical costs, which are distorted in favor of labor-intensive services, severely undercompensating these services (39). This historical cost-based pricing results in “cross-subsidization” during the formation of DIP points, resulting in point devaluation in secondary hospitals.

Different hospital levels in China continue to vigorously compete to maximize profits under the DIP system, intensifying competition within the regional medical market due to the point competition mechanism (6). Our findings show that public and tertiary hospitals exhibit superior cost control compared with private and secondary hospitals. Meanwhile, non-teaching hospitals outperform teaching hospitals in cost efficiency. This finding aligns with existing research indicating that while cost-shifting exists post-PPS implementation, it is not uniformly observed across all hospital types (40). Specifically, our study observed notable cost-shifting in secondary, public, and teaching hospitals, but it was less significant in tertiary, private, and non-teaching hospitals. Additionally, the primary pathway for cost-shifting involved shifting costs to copayments for category B rather than category C.

First, tertiary hospitals demonstrate more effective cost control than secondary hospitals. This finding is consistent with findings from Ding and Coelen, which indicated that hospitals with higher initial costs per case made more significant reductions compared with those with lower costs at the onset of PPS (41). In China, tertiary hospitals incur higher costs per hospitalization and handle a larger volume of services, including more complex surgical treatments than secondary hospitals, which have substantial unused bed capacities (42). The uncertainty of unit prices per point under DIP until year-end incentivizes tertiary hospitals to minimize costs to maximize revenue from insurance.

Second, non-teaching hospitals more effectively manage costs than teaching hospitals. DIP pricing fails to account for the additional costs associated with teaching and new technologies and projects that are more prevalent in teaching hospitals, resulting in higher uncompensated costs. Moreover, teaching hospitals, which are less efficient due to dual roles in education and patient care, face less pressure from patient acquisition due to their established market dominance. The more detailed disease grouping under DIP, compared with DRG, does not sufficiently incentivize cost control in teaching hospitals because it does in research-focused settings, according to Scanlon (43).

Third, public hospitals have significantly reduced the THE per case for CHD inpatients, contrasting with the non-significant increase observed in private hospitals. The operational goals of non-profit and for-profit hospitals differ, with private hospitals more focused on maximizing revenue. Post-DRG reform studies indicate that private hospitals engage in more intense coding practices than public hospitals and often transfer less profitable DRG patients to public hospitals (44, 45).

### 6.1 Limitations

Our study has four limitations. First, we did not assess how the DIP reform affected the number of hospital admissions due to constraints in data availability. In China’s competitive healthcare system, the point competition mechanism of DIP payments may influence regional budget distributions. Future research should explore the quantitative impacts of DIP payments on hospital admission rates. Second, the DIP payment reform has only been implemented for a short period, limiting our study to examining its effects over 15 months. Evidence from the Netherlands indicates that the long-term effects of DRG reforms can be more significant than the short-term effects (46, 47). Continuous monitoring of the DIP’s cost-containment and cost-shifting effects will be essential as the reform matures. Third, our analysis focused solely on the cost-containment and cost-shifting effects related to CHD. Hospitals may respond differently to various diseases. Future studies should compare the effects of DIP across different types of diseases, including surgical versus medical diseases and complex versus simple diseases. Fourth, we utilized logistic regression to compute the propensity scores; however, this method is constrained by its reliance on parametric modeling assumptions. Future research should explore the Super Learner machine learning algorithm for estimating propensity scores, as it may reduce this dependence and improve the robustness of the findings.

## 7 Conclusion

In summary, the DIP payment reform exhibits significant cost-containment and cost-shifting effects for CHD inpatients. On the insurance side, the reform substantially reduces the THE per case for CHD inpatients. On the patient side, the reform shifts costs from in-policy expenditures to IPER, with a greater shift intensity in the proportion of Class B compared to the proportion of Class C. However, the effects of cost-containment and cost-shifting significantly vary across different hospital categories, teaching statuses, hospital grades, and ownership.

## Data Availability

The data analyzed in this study is subject to the following licenses/restrictions: the data that support the findings of this study are available from Zunyi Local Health Security Administration but restrictions apply to the availability of these data, which were used under licence for the current study, and so are not publicly available. However, data are available from the authors upon reasonable request and with permission of Zunyi Local Health Security Administration. Requests to access these datasets should be directed to tanhuawei-2009@163.com.
